# Supplementary material for: Microwave Control of Reynoutria japonica Houtt., Including Ecotoxicological Aspects and the Resveratrol Content in Rhizomes
Source: Plants (Basel). 2024 Jan 5;13(2):152. doi: 10.3390/plants13020152 (PMC10818956; doi:10.3390/plants13020152)
Supplement: Supplementary file 1 [file plants-13-00152-s001.zip › plants-2721228-supplementary.pdf]

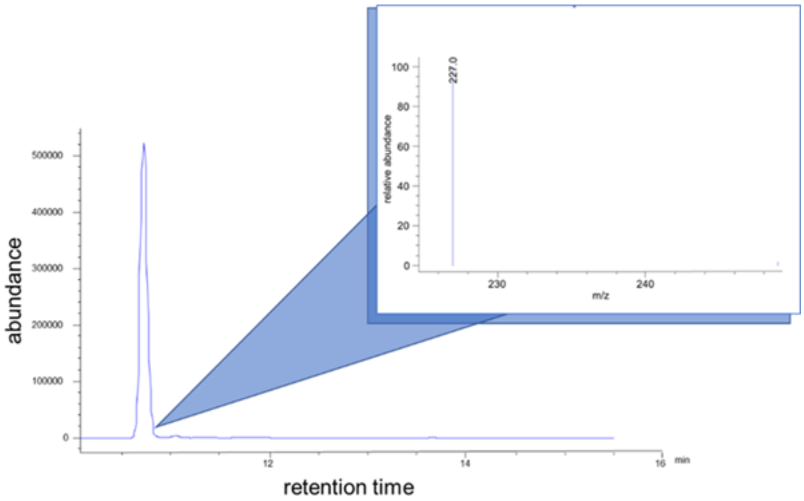

Figure S1. Extracted ion chromatogram (EIC) 227 with a relative mass spectrum of the trans-resveratrol peak.
